# Epidemiology of cancer in Saudi Arabia thru 2010–2019: a systematic review with constrained meta-analysis

**DOI:** 10.3934/publichealth.2020053

**Published:** 2020-09-11

**Authors:** Wedad Saeed Alqahtani, Nawaf Abdulrahman Almufareh, Dalia Mostafa Domiaty, Gadah Albasher, Manal Abduallah Alduwish, Huda Alkhalaf, Bader Almuzzaini, Salma Sanhaat AL-marshidy, Rgya Alfraihi, Abdelbaset Mohamed Elasbali, Hussain Gadelkarim Ahmed, Bassam Ahmed Almutlaq

**Affiliations:** 1 Faculty of Biology, Princess Nourah bint Abdulrahman University, Saudi Arabia; 2 Department of Pediatric Dentistry and Preventive Dental Sciences, Riyadh Elm University, Riyadh, Saudi Arabia; 3 Department of Biology, College of Science, Jeddah University, Jeddah, Saudi Arabia; 4 King Saud University, Department of Zoology, College of Science, Saudi Arabia; 5 Prince Sattam bin Abdulaziz University, College of Science and Humanities, Biology Department, Alkarj, Saudi Arabia; 6 King Faisal Specialist Hospital and Research Centre, Riyadh, Saudi Arabia; 7 King Abdullah International Medical Research Center, King Saud bin Abdulaziz University for Health Sciences, Ministry of National Guard Health Affairs, Riyadh, Saudi Arabia; 8 Department of Pharmaceutics, College of Pharmacy, King Saud University; 9 Pharm B, Pharmacy Services, Security Forces Hospital, Riyadh, Saudi Arabia; 10 Department of Clinical Laboratory Sciences, College of Applied Medical sciences, Jouf University, Qurayyat, Saudi Arabia; 11 College of Medicine, University of Hail, Saudi Arabia; 12 Department of Histopathology and Cytology, FMLS, University of Khartoum, Sudan

**Keywords:** cancer, Saudi Arabia, breast cancer, colon-rectal cancer, risk factors, meta-analysis

## Abstract

**Background:**

Cancer is emerging as a major global health-care system challenge with a growing burden worldwide. Due to the inconsistent cancer registry system in Saudi Arabia, the epidemiology of cancer is still dispersed in the country. Consequently, this review aimed to assemble the epidemiological metrics of cancer in Saudi Arabia in light of the available published data during the period from (2010–2019).

**Methods:**

Published literature from Saudi Arabia relating to cancer incidence, prevalence, risk factors, and other epidemiological metrics were accessed through electronic search in Medline/PubMed, Cochrane, Scopus, Web of Knowledge, Google Scholar, and public database that meet the inclusion criteria. Relevant keywords were used during the electronic search about different types of cancers in Saudi Arabia. No filters were used during the electronic searches. Data were pooled and odds ratios (ORs) and 95% confidence interval (95%CI) were calculated. A random-effects meta-analysis was performed to assess the well-determined risk factors associated with different types of cancers.

**Results:**

The most common cancers in Saudi Arabia are breast, colorectal, prostate, brain, lymphoma, kidney and thyroid outnumbering respectively. Their prevalence rates and OR (95%CI) as follow: breast cancer 53% and 0.93 (0.84–1.00); colon-rectal cancer (CRC) 50.9% and 1.2 (0.81–1.77); prostate cancer 42.6% and 3.2 (0.88–31.11); brain/Central Nervous System cancer 9.6% and 2.3 (0.01–4.2); Hodgkin and non-Hodgkin's lymphoma 9.2% and 3.02 (1.48–6.17); kidney cancer 4.6% and 2.05 (1.61–2.61), and thyroid cancer 12.9% and 6.77 (2.34–19.53).

**Conclusion:**

Within the diverse cancers reported from Saudi Arabia, the epidemiology of some cancers magnitude 3-fold in the latest years. This increase might be attributed to the changing in the Saudi population lifestyle (adopting western model), lack of cancer awareness, lack of screening & early detection programs, social barriers toward cancer investigations. Obesity, genetics, sedentary lifestyle, tobacco use, viral infection, and iodine & Vit-D deficiency represent the apparent cancer risk factors in Saudi Arabia.

## 1. Introduction

Cancer is responsible for more than 9.6 million deaths in about 185 countries, ranked as the second leading cause of mortality worldwide [1].

Many risk factors have been implicated in the etiology of cancer including; tobacco and alcohol consumption, unhealthy diet, physical inactivity, viral infection, bacterial infection, urban air pollution, ionizing radiation and indoor smoke [1],[2]. It is expected that, due to changes in population demographics in the next decades, cancer will continue rising to 21.4 million deaths worldwide, by 2030 [3]. Overall cancer burden, as well as, increased survival rates can be achieved through cancer prevention, early detection strategies [4].

In 2018, there 10518 cancer deaths with 24,485 new cancer cases in Saudi Arabia (total population = 33,554,333) [5]. The most common cancers include breast cancer, colon-rectum (CRC), and prostate [6]. Frequently reported risk factors associated with breast cancer were hormonal variations, diet, lifestyle, and obesity [7]. Recent researches reported increasing trends of CRC in Saudi Arabia [8],[9]. In 2018, CRC accounts for 14.6% of total cancers in the country [5]. The risk factors for CRC may be genetic, environmental, age, gender and other inflammatory conditions of the digestive tract [10],[11].

Prostate cancer is the other major cause of death in males. Alteration in lipid metabolism, HPV infection and racial difference are some of the risk factors linked with prostate cancer [12].

As per the Saudi cancer registry, the prevalence of brain cancer is comparatively low in Saudi Arabia accounting for only 2.0 to 3.2% in females and males respectively [13]. Like other types of cancers, central nervous system (CNS) tumors are also gaining momentum worldwide and ranks among the top 10 mortalities due to cancers. One of the possible risk factors for CNS tumors is radioactive exposure [14]. This in addition to the metastasis, particularly from breast cancer [15].

Lymphomas Hodgkin's (HL) and non-Hodgkin's (NHL), extranodal non-Hodgkin lymphoma (EN-NHL)) are other common malignancies affecting both young and adult Saudi [16]–[19]. The prevalence of HL (uncommon type) was reported as 3.4% in Saudi Arabia with high clinical variability and frequently observed among the age-range 15–35 years [20]–[22].

Kidney cancer is the other more commonly diagnosed cancer in Saudi Arabia. The tendency is towards the increasing trend. According to the cancer registry in 2013, the age-standardized rate of kidney cancer was 2.3% of all cancers. In recent decades, the incidence is increasing due to various risk factors [23]. The common risk factors associated with kidney cancer are smoking, obesity, diabetes, and hypertension [24]. The common histological type in renal cell carcinoma is clear cell carcinoma [25].

Thyroid carcinoma is one of the frequently diagnosed thyroid disorders [26]. The incidence is more common among females compared to males. The prevalence of thyroid cancer 10.1% [5] and the common malignant type being papillary thyroid carcinoma [27].

**Figure 1.**
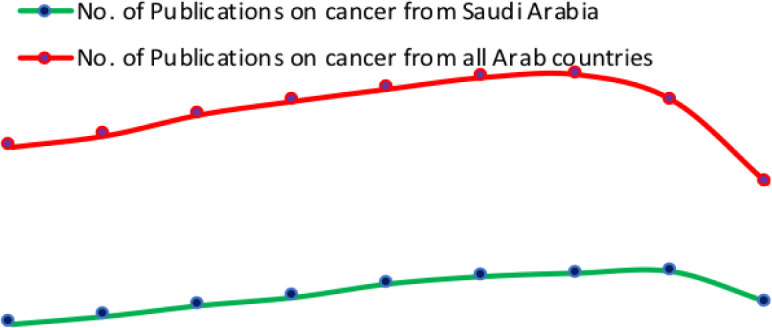
Cancer-related publications from Saudi Arabia vs. all Arab countries.

Interestingly, in recent years, there were submerging of cancer-related literature (5,000 articles in 2010 to 11,000 in 2017) from Saudi Arabia compared to neighboring Arab countries, as indicated in Figure 1. So this review aimed to assemble the epidemiological metrics of cancer in Saudi Arabia in light of the available published data during the period from (2010–2019).

## 2. Methodology

### 2.1. Data sources and search strategy

Literature from Saudi Arabia related to various cancer types were collected by electronic search in Medline/PubMed, Cochrane Library, Scopus, Web of Knowledge, Google Scholar and public database (GLOBOCAN 2012, IARC) that meet the inclusion criteria. Data from the Saudi cancer registry were also collected. Relevant keywords (breast cancer, colon-rectum (CRC), prostate, Hodgkin's and non-Hodgkin's lymphomas, brain/CNS, kidney, thyroid, etc.) were used in affiliation to Saudi Arabia. No filters were used during the electronic searches.

### 2.2. Selection of required publications

**Figure 2.**
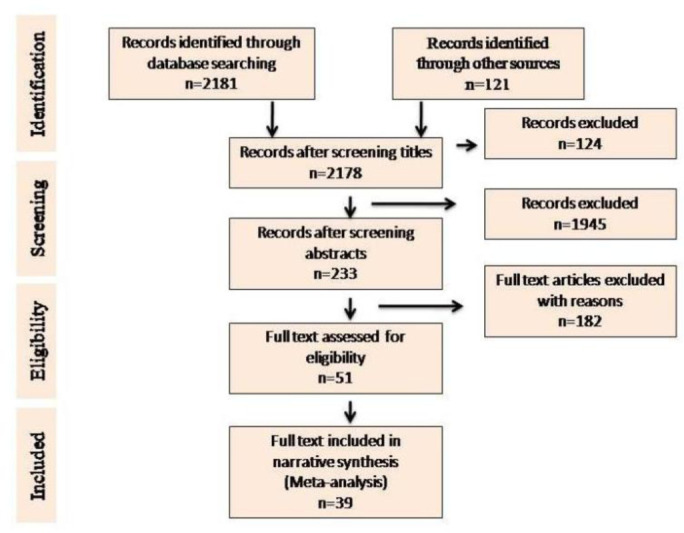
Flow chart for the selection process of articles included in the review.

In-depth selections were made through search engines following effective inclusion and exclusion criteria (Figure 2).

#### 2.2.1. Inclusion criteria

Only literature published during 2010–2019 from Saudi Arabia and related to the epidemiology of cancer and risk factors were included. Data from the Saudi National cancer registry were also considered. All sources which demonstrate the type of malignant neoplasm (C18-21, C-50, C-61, C64-66, C70-72, C73, and C81-85), with possible risk factors were put under inclusion criteria. All relevant articles (including case-control, cohort, cross-sectional, etc.) were included, as they permit the estimation of odds ratios (OR) and 95% confidence intervals (95%CI).

#### 2.2.2. Exclusion criteria

Publications are written in a language other than English and those focussed on survivors of cancer, pharmacological research, qualitative studies, and reviews and meta-analysis were excluded. Publications on laboratory research including animal trials were also excluded.

### 2.3. Quality appraisal

After scanning the titles of all relevant publications and reading the abstracts of the selected publications full-text papers were appraised by the assigned reviewers using PRISMA guidelines [28].

### 2.4. Assessment of heterogeneity and statistical parameters

Valid statistical package (Comprehensive Meta-analyses ver. 3) was used to calculate the summary effect estimate and 95% confidence intervals to test for heterogeneity of prevalence and risk factors estimation. Heterogeneity, as determined using the I2 index statistic, was classified into low (scores <25%) and high (scores ≥75%) heterogeneity. DerSimonian-Laird random-effects meta-analysis was used to summarize the prevalence of different cancer types with possible risk factors. Minimum three independent studies were taken into account for summary and subgroup analysis to justify the analysis. Only in one analysis, due to the paucity of data for brain/CNS cancer from Saudi Arabia, two independent studies were used. Statistical package (SPSS ver. 25) were used and p-values <0.05 were considered statistically significant.

## 3. Results and discussions

### 3.1. Search results

The systematic database search yielded 2,181 records and 121 additional records from other sources. After excluding 124 records, a total of 2,178 records were further screened. After several selection processes, 39 full-text records were included for meta-analysis (Figure 2).

### 3.2. Epidemiology

**Figure 3.**
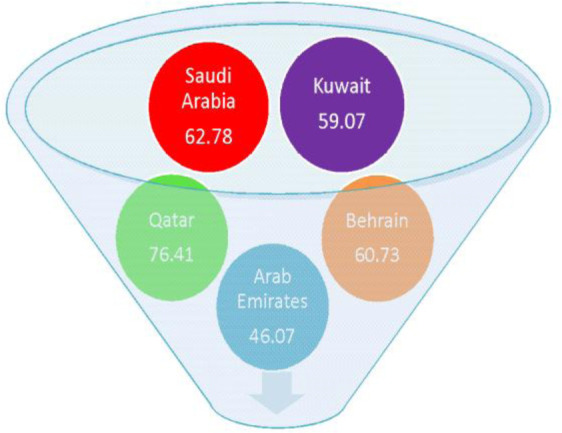
Estimated death rates/100,000 due to cancer in Saudi Arabia in comparison to Arabian Gulf countries (WHO 2017).

With regard to age-standardized rates (ASR), the incidence of all cancers excluding non-melanoma skin cancer was estimated to be 17,522 cases (8,296 males and 9,226 females) in Saudi Arabia with ASR incidence rates of 0.3–12.6 in males and 0.2–29.5 in females. The estimated mortality was 9,134 cases across all ages and gender [29]. The ASR mortality rates range from 0.1–7.3 in males and 0.1–9.1 in females. Saudi Arabia ranks second in cancer mortality rates amongst all Arabian Gulf countries (Figure 3).

**Figure 4.**
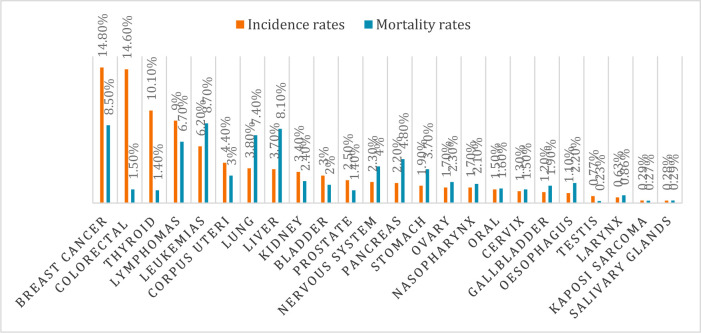
Incidence and mortality rates in Saudi Arabia in 2018.

**Figure 5.**
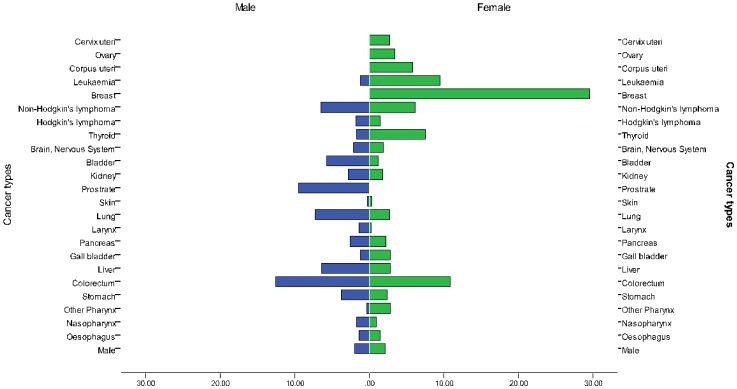
Estimated age-standardized incidence rates of cancers in Saudi Arabia per 100,000 (Source: WHO 2017).

Breast cancer is leading cancer in Saudi Arabia with an incidence and mortality rates of 14.8% (cumulative risk 2.87%) and 8.5%(cumulative risk 0.81%) among both sexes, respectively. In 2018 the incidence of breast cancer among females was 29.7% in Saudi Arabia. Colorectal cancer ranked the third most common cancer in Saudi Arabia with an incidence and mortality rate of 14.6% (cumulative risk 1.47%) and 1.48%(cumulative risk 0.65%) among both sexes, respectively. The incidence among males was 19.6% and females 9.5%. Thyroid cancer ranked the third most common cancer in Saudi Arabia with an incidence and mortality rate of 10.1% (cumulative risk 0.67%) and 1.4% (cumulative risk 0.07%) among both sexes, respectively. The incidence was high among females 14.1% compared to 6% among males [5], as shown in Figure 4 & 5.

Table 1 provides a summary of the epidemiological indicators as measured in the included studies, viz., incidence, and prevalence, mean age at diagnosis, malignant sites.

**Table 1.** Incidence, prevalence and possible risk factors of some common and rare cancers in Saudi Arabia.

Study parameters	Source of data	Period	No. of cases & control/biopsies under study (n)	Age group	Common Malignant sires	Risk factor distribution among patients	Reference
Case-control	Hospital-based	2009	200	50 ± 5	Breast	Low intake of folate, MTHFR polymorphism	Alshatwi 2010
Case-control	Hospital-based	2009–2010	200	50 ± 5	Breast	TP 53 & MDM polymorphism	Alshatwi et al. 2011
Case-control	Hospital-based	NS	208	17–80	Breast	P53 codon 72 polymorphism	Al-Qasem et al. 2012
Case-control	Hospital-based	NS	189	NS	Breast	Micro RNA polymorphism	Alshatwi et al. 2012
Retrospective study	Cancer registry	2001–2008	6922	30–59	Breast	NS	Alghamdi et al. 2013
Case-control	Hospital-based	NS	195	~48	Breast	PARP-1V762A polymorphism	Alanazi et al. 2013
Case-control	Hospital-based	2009	240	18–75	Breast	Vitamin-D deficiency	Yousef et al. 2013
Case-control	Hospital records	NS	200	~48	Breast	XRCC1 polymorphism	Al-Mutairi et al. 2013
Case-control	Hospital-based	NS	109	9–47	Breast	Obesity	Alokali et al. 2013
Case-control	Hospital-based	2010–2011	200	37–61	Breast	BRCA1 & BRCA2 gene polymorphism	Hasan et al. 2013
Case-control	Hospital-based	2007–2012	1172	~35	Breast	Obesity	Elkum et al. 2014
Cross-sectional	Hospital-based	2011–2013	801	42–57	Breast	Low vitamin D levels	Abulkhair et al. 2015
Case-control	Hospital-based	2001–2013	192	30–65	Breast	Oral contraceptive, abortion	Karim et al. 2015
Case-control	Hospital-based	NS	200	~40	Breast	VEGF-gene variation	Al Balawi et al. 2018
Case-control	Hospital-based	NS	200	~40	Breast	Genetical	Mir et al. 2018
Case-control	Hospital-based	2009	120	50–67	Colon	Serum resistin	Al-Harithy & Al-Ghafari 2010
Case-control	Hospital-based	NS	120	50–65	Colon	Genetic polymorphism of the ADIPOQ gene	Al-Harithy & Al-Zahrini 2012
Case-control	Hospital-based	2008–2010	130	45–80	Colon	Polymorphism of XRCC1	Al-Harithy & Al-Ghazzawi 2011
Case-control	Hospital-based	NS	120	NS	Colon	Polymorphism of RETN gene	Al-Harithy 2014
Case-control	Hospital-based	2013–2014	200	20–80	Colorectal	Vitamin D receptor (VDR) polymorphism	Alkhayal et al. 2016
Prospective	Hospital-based	2010–2015	280	~51	Colorectal	Family history, ulcerative colitis, fatty diet	Aldiab 2017
Case-control	Hospital-based	2006–2015	297	29–60	Colorectal	Family history, Lynch syndrome, Gene variant	Alqahtani et al. 2017
Retrospective	Hospital records	NS	572	<50 to >70	Prostate	The racial difference, high serum PSA	Al-Abdin 2013
Retrospective	Hospital records	2006–2013	417	20–95	Prostate	NS	Albasri et al. 2014a
Retrospective	Hospital records	2001–2008	1739	0 to ≥75	Prostate	NS	Alghamidi et al. 2014
Retrospective	Hospital records	2010–2015	291	0–65	Brain	NS	Taha et al. 2018
Cross-sectional	NS	2015–2016	1500	<18 to >40	CNS	Radioactive occupation, genetical, low physical activity	Aljuhani et al. 2018
Retrospective	Hospital records	1997–2012	340	25–82	Hodgkin's lymphoma	EBV virus, genetical, environmental factors	Shafi et al. 2017
Retrospective	Hospital records	1983–2003	83	<10–80	Non-Hodgkin's lymphoma (EN-NHL)	NS	Nagi et al. 2010
Retrospective	Hospital records	1994–1999	1209	14–60	Non-Hodgkin's lymphoma (EN-NHL)	Genetical, environmental	Al Diab et al. 2011
Retrospective	Hospital records	2006–2013	346	3–96	Non-Hodgkin's lymphoma	NS	Albasri et al. 2014b
Retrospective	Hospital records	1990–2010	382	≥18	Kidney	Smoking, diabetes, obesity, hypertension	Alkhateeb et al. 2015
Retrospective	Hospital records	1990–2015	371	45–65	Kidney	Smoking, diabetes, obesity, hypertension, dyslipidemia, incidental	Alkhateeb et al. 2018
Retrospective	Hospital records	2003–2013	219	22–95	Kidney	NS	Mahasin et al. 2018
Retrospective	Hospital records	1998–2007	668	50–60	Thyroid	NS	Rafeidi et al. 2010
Retrospective	Hospital records	2000–2010	2292	39(mean)	Thyroid	Iodine deficiency, family history, high leptin level, radiation exposure	Hussain et al. 2013
Retrospective	Hospital records	2006–2013	292	<20 to >60	Thyroid	Iodine deficiency	Albasri et al. 2014
Retrospective	Hospital records	2008–2010	312	<20 to >60	Thyroid	NS	Saeed et al. 2018
Retrospective	Hospital-based	2015	168	19–23	Thyroid	NS	Bafaraj et al. 2018

Note: NS—Not specified.

### 3.3. Etiology and meta-analysis

Although the incidence of cancer, particularly breast cancer is high in high-income countries, mortality is high in low or medium-income countries [30]. The polymorphism in BRCA1-3’UTR and VEGF gene are reported to be strong genetic factors linked to breast cancer [31],[32]. Mutation in BRCA1 and BRCA2 genes are reported to increase the development of breast and ovarian cancers [33]. Many factors are associated with the risk of breast cancer including; the use of oral contraceptives, menstrual history, nulliparity obesity, family history of breast cancer, and low vitamin D [34],[35]. Early diagnosis, positive attitude, and awareness are some of the possible measures to mitigate the mortality rate due to breast cancer [36].

**Figure 6.**
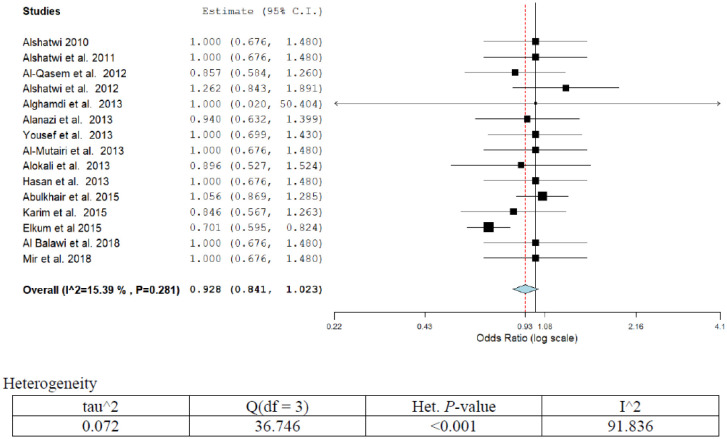
Meta-analyses (forest plot) of the prevalence of breast cancer with possible risk factors.

Overall fifteen publications reported different forms of risk factors associated with breast cancer (Table 1; Figure 6). The overall pooled breast cancer prevalence was 53% (95%CI; 42.2–47.5) including 14 studies between 2010 to 2018. The prevalence increased over time significantly in Saudi Arabia (*p* < 0.002). Nine publications reported OR that has been adjusted for gene variations and other genetic parameters. Three publications have reported on low vitamin D intake and the other three on obesity. The adjusted OR and 95%CI ranged from 0.95 (0.64–1.4) to 1.3 (0.85–2.0) for gene variations or polymorphism [37]–[45],[32],[31]. The OR for low intake of folate or vitamin D ranged from 1.00 (0.87–1.3) to 1.00 (0.70–1.50) [43],[35]. The OR (95%CI) for obesity as a risk factor for breast cancer ranged from 0.70 (0.60–0.83) to 0.90 (0.53–1.53) [44],[46]. Only one publication highlighted the use of oral contraceptives or abortion as possible risk factors with OR (95%CI) of 0.85 (0.57–1.3). The overall pooled estimate was OR (95%CI) = 0.93 (0.84–1.0). A low degree of heterogeneity was observed in the present analysis (I^2^ = 15.39%, *p* = 0.29). After eliminating low-quality studies (scoring ≤3 points out of 8), a sensitivity analysis was conducted through repeating meta-analysis, and however, no significant alteration in results was observed.

A high risk of CRC was observed in the age range of 35–65 years. The common risk factors for CRC are inflammatory bowel disease, smoking, and family history, in addition to environmental and genetic factors. Screening at regular intervals using colonoscopy, sigmoidoscopy and fecal immunochemical tests are recommended for CRC [47]. Some studies suggest that low vitamin D intake is associated with risk of colorectal cancer [48]. The prevalence of colon-rectum cancer was 50.9% (95%CI; 40.22–45.1) as revealed from pooled seven studies of this series. The prevalence significantly increased over time (*p* < 0.001).

**Figure 7.**
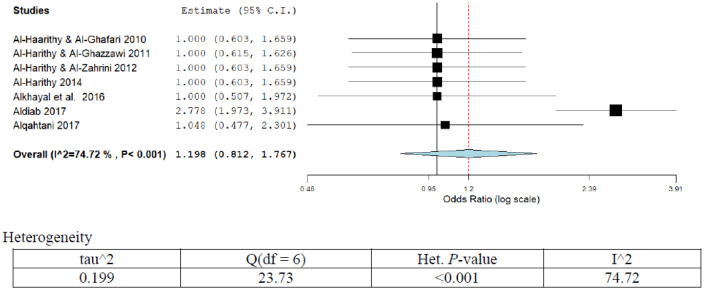
Meta-analyses (forest plot) of the prevalence of CRC with possible risk factors.

**Figure 8.**
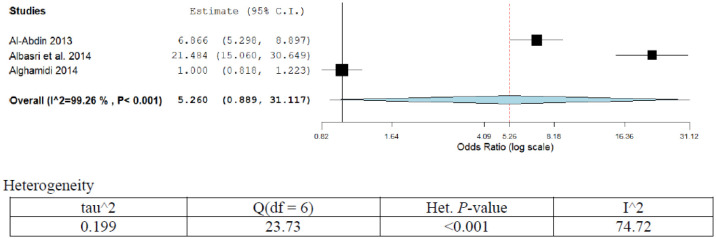
Meta-analyses (forest plot) of the prevalence of prostate cancer with possible risk factors.

Out of the overall seven publications reporting different forms of risk factors (Table 1; Figure 7), six studies reported genetic polymorphism as the possible risk factors. The adjusted OR (95%CI) ranged from 1.00 (0.50–2.00) to 1.00 (0.50–2.30) [11],[49]–[53]. Two studies have attributed family history as a possible risk for CRC, with OR (95%CI) ranged from 1.00 (0.50–2.3) to 2.8 (2.00–4.1) [8],[11]. Only one study has highlighted ulcerative colitis and fatty diet as possible risk factors for CRC with OR (95%CI) of 2.8 (2.0–2.4) [8]. The pooled OR (95%CI) for the overall risk factors was 1.2 (0.81–1.77). A high degree of heterogeneity was observed in the present analysis (I^2^ = 75%; *p* < 0.001). Sensitivity analysis showed a high pooled prevalence of genetic polymorphism positivity as reported by the studies (75%; 95%CI; 15.3–81.1%) and was found statistically significant (*p* < 0.001).

Prostate cancer is one of the common cancers in males with high morbidity and mortality in Saudi Arabia. It is more prevalent in the age group of 50–70 years. The prostatic disease may be benign prostatic hyperplasia or carcinoma prostrates [54]. Several risk factors were implicated in the etiology of prostate cancer [55]–[57].

The prevalence of prostate cancer was 42.6% (95%CI; 36.12–41.2) as revealed from pooled three studies in the present series (Table 1; Figure 8). The prevalence was statistically insignificant over time. The OR (95%CI) ranged from 1.00 (0.82–1.22) to 21.5 (15.0–30.6) [54],[57],[58]. Only one study has assigned racial differences and high serum prostate-specific antigen (PSA) as possible risk factors for prostate cancer, OR (95%CI) 6.9 (5.3–8.9). The overall pooled OR was 5.2 (0.89–31). A high degree of heterogeneity was observed in the present analysis (I^2^ = 99.26%; *p* < 0.001). Sensitivity analysis showed no significant variation in the results.

Tumors of the brain and central nervous system (CNS) differ from other tumors develop in various body tissues. Some of the signs and symptoms associated with CNS tumors are headaches, drowsiness, lack of awareness and increased sleep duration [14]. Some of the risk factors identified are exposure to radioactivity, bisphenol acetate, X-rays, increased body mass index and prolonged use of mobile phones. Vitamin D has been reported to prevent the progression of brain cancer by maintaining the integrity of the adhesion protein.

**Figure 9.**
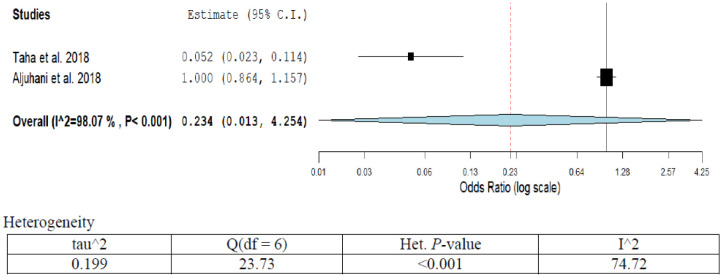
Meta-analyses (forest plot) of the prevalence of brain & CNS cancer with possible risk factors.

The prevalence of brain/CNS cancer was only 9.6% (95%CI; 11.10–16.2) as revealed from pooled studies this series (Table 1; Figure 9). The OR (95%CI) ranged from 0.05 (0.02–0.11) to 1.0 (0.9–1.2) [13],[14]. The overall pooled OR (95%CI) was 0.23 (0.01–4.2). The attributed risk factors were radioactive exposure, genetic and low physical activity. A high degree of heterogeneity was observed in the present analysis (I^2^ = 98.0%; *p* < 0.001). Sensitivity analysis showed no significant variation in the results.

The incidence rates of HL are comparatively low than the NHL in Saudi Arabia. The incidence of HL is more prevalent among young people of 15–35 years. However, it may recur after the age of 50 years. In one study from Saudi Arabia, the overall survival rate of patients with HL was found to be 91% [59]. Recent studies showed that infection with hepatitis C&B viruses is a common risk factor [60].

**Figure 10.**
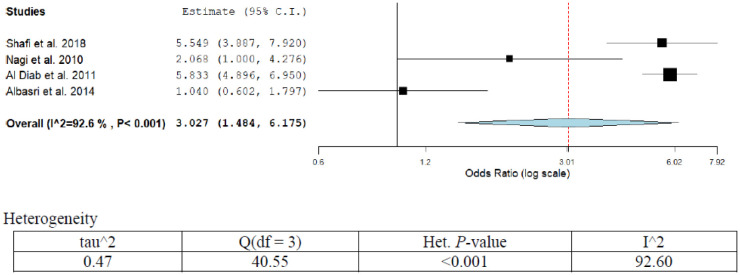
Meta-analyses (forest plot) of the prevalence of HL & NHL cancers with possible risk factors.

The prevalence of Hodgkin and non-Hodgkin lymphoma was 9.2% (95%CI; 10.1–15.6) as revealed from pooled four studies recruited in this series (Table 1; Figure 10). The pooled OR (95%CI) combining all studies was 3.02 (1.48–6.17). Prevalence was statistically significant over time (*p* < 0.05). The OR (95%CI) ranged from 1.0 (0.60–1.80) to 5.5 (3.97–7.92). Two studies have assigned genetic and Epstein-Barr viral infection as a possible risk factor for Hodgkin and non-Hodgkin lymphoma, OR (95%CI) 5.5 (3.9–8.0) and 5.8 (4.9–7.0) [19],[59]. A high degree of heterogeneity was observed in the present analysis (I^2^ = 92.6%; *p* < 0.001). Sensitivity analysis showed significant variation in the results (*p* < 0.05). The pooled prevalence was 26.1% (95%CI; 21.0–29.1%).

Kidney cancer is the commonest genitourinary tract malignancy [61]. Patients with hypertension, diabetes mellitus, and habitual smoking are more likely to have kidney cancer [24]. Obesity and dyslipidemia are other possible risk factors. Other reported risk factors include gender and age, with the incidence being more prevalent in males and the older age group [23].

**Figure 11.**
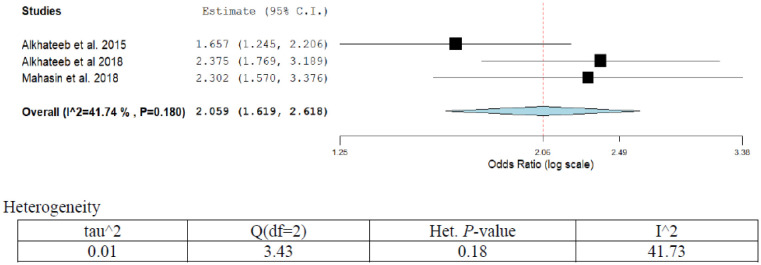
Meta-analyses (forest plot) of the prevalence of kidney cancer with possible risk factors.

Overall two publications out of total three, reported different forms of risk factors associated with kidney cancer (Table 1; Figure 11). The overall pooled kidney cancer prevalence was 4.6% (95%CI; 2.2–3.7) in the three studies in our series. Prevalence increased over time significantly in Saudi Arabia (*p* < 0.002). Two publications reported OR that has been adjusted for diabetes and obesity. The adjusted OR (95%CI) ranged from 1.65 (1.24–2.20) to 2.3 (1.57–3.76) for diabetes and obesity [24],[61]. The overall pooled estimate was OR (95%CI) = 2.05 (1.61–2.61). A medium degree of heterogeneity was observed in the present analysis (I^2^ = 41.74%; *p* = 0.18). After eliminating low-quality studies (scoring ≤3 points out of 8). Sensitivity analysis was conducted through repeating meta-analysis, however, the results showed no significant alteration.

**Figure 12.**
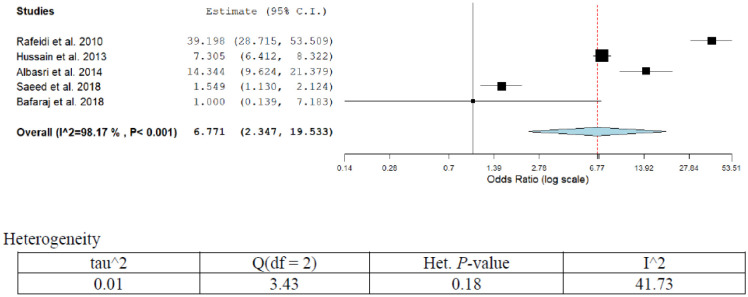
Meta-analyses (forest plot) of the prevalence of thyroid cancer with possible risk factors.

Population-based studies have revealed an increasing trend of thyroid cancer in the past few decades. The disease varies from region to another. The commonest existing histological subtype is papillary thyroid carcinoma [62]. The incidence of thyroid cancer increases with the increase of age. Radiation exposure of the thyroid gland is one of the possible risk factors of thyroid cancer [63]. Thyroid cancer is more prevalent in females compared to males with an incidence rate of 7.5%. Overall five publications reported different forms of risk factors associated with thyroid cancer (Table 1; Figure 12). The overall pooled thyroid cancer prevalence was 12.9% (95%CI; 10.2–11.7) in five studies of our series. Two publications reported OR that has been adjusted for iodine deficiency. The adjusted OR (95%CI) ranged from 1.0 (0.13–7.1) to 39.0 (28.7–53.5) for iodine deficiency [64],[65]. The other risk factors highlighted in the studies are radiation exposure and high leptin level. The overall pooled estimate was 6.77 (2.34–19.53). A high degree of heterogeneity was observed in the present analysis (I^2^ = 98.17%; *p* < 0.001). After eliminating low-quality studies (scoring ≤3 points out of 8). Sensitivity analyses were conducted by repeating meta-analysis. Iodine deficiency reported by studies showed medium sensitivity (42.3%; 95%CI; 19.4–55.1%).

The highly recognized risk factors for cancer as revealed by most of the literatures are lack of lifestyle healthy habit, obesity, low vitamin D intake, use of oral contraceptives, abortion, ulcerative colitis, genetic polymorphism, high serum PSA, radioactive exposure, low physical activity, Epstein-Barr viral infection, diabetes, iodine deficiency, smoking, and high leptin levels. Besides these, ambient air pollution is another risk factor for most of the malignancies in Saudi Arabia due to frequent exposure to dust storms containing particulate matters [66]. However, the present study predict that, almost all meta-analyses were highly heterogeneous, which might be attributed to the diverse cancer causes factors, lack of the studies, and intermittence of cancer reporting registries.

### 3.4. Miscellaneous factors

Within the context of literature pertained to observational studies (assessing perception, attitude, and awareness), many Saudi community-related factors can contribute to the escalating burden of cancer. These increasing trends may be due to changing lifestyles, lack of awareness, embarrassment, fear of testing or non-accessibility to advanced treatment and due to a multitude of factors. Although most of these cancers are preventable, early detection by trained health practitioners is of utmost importance and paramount responsibility. Effective techniques for screening and diagnosis using modern up-graded instruments may minimize the burden of cancer in Saudi Arabia [10],[67]–[69]. The present review has direct implications in improving the health component of cancer patients through the inspiration of health providers towards implementing effective cancer-related health management strategies. These should include; prevention, early detection, proper treatment, and better palliative care. Such strategies will recruit a healthy lifestyle, raised awareness (decreasing cancer morbidity), early detection resulting, appropriate treatment, and better palliative care (decrease mortality).

## 4. Conclusion

Within the diverse cancers reported from Saudi Arabia, the epidemiology of some cancers magnitude 3-fold in the latest years. This increase might be attributed to the changing in the Saudi population lifestyle (adopting western model), lack of cancer awareness, lack of screening & early detection programs, social barriers toward cancer investigations. Obesity, genetics, sedentary lifestyle, tobacco use, viral infection, and iodine & Vit-D deficiency represent the apparent cancer risk factors in Saudi Arabia.
